# Fulminant corticobasal degeneration: a distinct variant with predominant neuronal tau aggregates

**DOI:** 10.1007/s00401-019-02119-4

**Published:** 2020-01-16

**Authors:** Helen Ling, Ellen Gelpi, Karen Davey, Zane Jaunmuktane, Kin Y. Mok, Edwin Jabbari, Roberto Simone, Lea R’Bibo, Sebastian Brandner, Matthew J. Ellis, Johannes Attems, David Mann, Glenda M. Halliday, S. Al-Sarraj, J. Hedreen, James W. Ironside, Gabor G. Kovacs, E. Kovari, S. Love, Jean Paul G. Vonsattel, Kieren S. J. Allinson, Daniela Hansen, Teisha Bradshaw, Núria Setó-Salvia, Selina Wray, Rohan de Silva, Huw R. Morris, Thomas T. Warner, John Hardy, Janice L. Holton, Tamas Revesz

**Affiliations:** 1 Queen Square Brain Bank for Neurological Disorders, UCL Queen Square Institute of Neurology, University College London, 1 Wakefield Street, London, WC1N 1PJ UK; 2 Reta Lila Weston Institute for Neurological Studies, Department of Clinical and Movement Neurosciences, UCL Queen Square Institute of Neurology, University College London, London, UK; 3 Department of Neurodegenerative Disease, UCL Queen Square Institute of Neurology, University College London, London, UK; 4 Neurological Tissue Bank of the Biobanc-Hospital Clinic-IDIBAPS, Barcelona, Spain; 5 Institute of Neurology, Medical University of Vienna, Vienna, Austria; 6 Division of Neuropathology, National Hospital for Neurology and Neurosurgery, University College London Hospital Trust, Queen Square, London, UK; 7 UK Dementia Research Institute, Department of Neurodegenerative Disease, UCL Queen Square Institute of Neurology, University College London, London, UK; 8 Division of Life Science, Institute for Advanced Study, Hong Kong University of Science and Technology, Hong Kong Special Administrative Region, Hong Kong, China; 9 Cancer Sciences Unit, University of Southampton, Southampton, UK; 10 Newcastle Brain Tissue Resource, Institute of Neuroscience, Newcastle University, Newcastle, UK; 11 Manchester Brain Bank, University of Manchester, Manchester, UK; 12 Sydney Brain Bank, Neuroscience Research Australia (NeuRA), Sydney, Australia; 13 Brain and Mind Centre and Central Clinical School, Faculty of Medicine and Health, University of Sydney, Sydney, Australia; 14 The London Neurodegeneration Brain Bank, The Institute of Psychiatry Psychology and Neurosciences (IOPPN), Kings College London, London, UK; 15 The Harvard Brain Tissue Resource Centre, McLean Hospital, Belmont, USA; 16 National CJD Research and Surveillance Unit, Centre for Clinical Brain Sciences, University of Edinburgh, Edinburgh, UK; 17 University of Toronto, University Health Network, and Tanz Centre for Research in Neurodegenerative Disease, Toronto, Canada; 18 Department of Psychiatry, HUG Belle-Idée, University of Geneva School of Medicine, Geneva, Switzerland; 19 South West Dementia Brain Bank, University of Bristol, Bristol, UK; 20 Taub Institute for Research on AD and the Aging Brain, Columbia University Medical Center, New York, USA; 21 Cambridge Brain Bank, Cambridge University Hospitals, Cambridge, UK

**Keywords:** Corticobasal degeneration, Astrocytic plaques, Progressive supranuclear palsy, Neurofibrillary tangles, Tau

## Abstract

Corticobasal degeneration typically progresses gradually over 5–7 years from onset till death. Fulminant corticobasal degeneration cases with a rapidly progressive course were rarely reported (RP-CBD). This study aimed to investigate their neuropathological characteristics. Of the 124 autopsy-confirmed corticobasal degeneration cases collected from 14 centres, we identified 6 RP-CBD cases (4.8%) who died of advanced disease within 3 years of onset. These RP-CBD cases had different clinical phenotypes including rapid global cognitive decline (*N* = 2), corticobasal syndrome (*N* = 2) and Richardson’s syndrome (*N* = 2). We also studied four corticobasal degeneration cases with an average disease duration of 3 years or less, who died of another unrelated illness (Intermediate-CBD). Finally, we selected 12 age-matched corticobasal degeneration cases out of a cohort of 110, who had a typical gradually progressive course and reached advanced clinical stage (End-stage-CBD). Quantitative analysis showed high overall tau burden (*p* = 0.2) and severe nigral cell loss (*p* = 0.47) in both the RP-CBD and End-stage-CBD groups consistent with advanced pathological changes, while the Intermediate-CBD group (mean disease duration = 3 years) had milder changes than End-stage-CBD (*p* < 0.05). These findings indicated that RP-CBD cases had already developed advanced pathological changes as those observed in End-stage-CBD cases (mean disease duration = 6.7 years), but within a significantly shorter duration (2.5 years; *p* < 0.001). Subgroup analysis was performed to investigate the cellular patterns of tau aggregates in the anterior frontal cortex and caudate by comparing neuronal-to-astrocytic plaque ratios between six RP-CBD cases, four Intermediate-CBD and 12 age-matched End-stage-CBD. Neuronal-to-astrocytic plaque ratios of Intermediate-CBD and End-stage-CBD, but not RP-CBD, positively correlated with disease duration in both the anterior frontal cortex and caudate (*p* = 0.02). In contrast to the predominance of astrocytic plaques we previously reported in preclinical asymptomatic corticobasal degeneration cases, neuronal tau aggregates predominated in RP-CBD exceeding those in Intermediate-CBD (anterior frontal cortex: *p* < 0.001, caudate: *p* = 0.001) and End-stage-CBD (anterior frontal cortex: *p* = 0.03, caudate: *p* = 0.01) as demonstrated by its higher neuronal-to-astrocytic plaque ratios in both anterior frontal cortex and caudate. We did not identify any difference in age at onset, any pathogenic tau mutation or concomitant pathologies that could have contributed to the rapid progression of these RP-CBD cases. Mild TDP-43 pathology was observed in three RP-CBD cases. All RP-CBD cases were men. The *MAPT* H2 haplotype, known to be protective, was identified in one RP-CBD case (17%) and 8 of the matched End-stage-CBD cases (67%). We conclude that RP-CBD is a distinct aggressive variant of corticobasal degeneration with characteristic neuropathological substrates resulting in a fulminant disease process as evident both clinically and pathologically. Biological factors such as genetic modifiers likely play a pivotal role in the RP-CBD variant and should be the subject of future research.

## Introduction

Corticobasal degeneration (CBD) typically has a slow progressive course with a disease duration ranging from 5 to 7 years [3]. Autopsy-confirmed CBD cases with disease duration of 3 years or less are rare but have been described in the literature. A report described a CBD patient who presented with a 10-month history of rapidly progressive corticobasal syndrome described as ‘fulminant’ CBD [55]. In a small series of 14 CBD cases, one patient had a disease duration of 2.5 years [65], while another series of 15 CBD cases reported a patient who succumbed to the illness after 2 years [48].

A phenotypic presentation of corticobasal syndrome can be caused by CBD as well as a number of other pathologies [42]. On the other hand, clinical presentations of CBD are heterogeneous and were categorised by the latest clinical diagnostic criteria into four main phenotypes [3]: corticobasal syndrome, frontal behavioural-spatial syndrome, non-fluent and agrammatic variant of primary progressive aphasia, and progressive supranuclear palsy syndrome or Richardson’s syndrome. These phenotypes encompass a constellation of cognitive, behavioural, language, motor and movement impairments. When a rapidly progressive course is encountered, some of these phenotypes may be encompassed under the umbrella term of ‘rapidly progressive dementias’ [19]. Prion disease, notably Creutzfeldt-Jakob disease, is a potential underlying diagnosis in rapidly progressive dementias [19]. Sporadic Creutzfeldt-Jakob disease presenting with a pure clinical phenotype of corticobasal syndrome or Richardson’s syndrome has also been reported. These cases typically had an aggressive and relentless clinical course leading to death in less than one year [4, 31, 38, 47, 52, 63]. Genetic Creutzfeldt-Jakob disease [45] and Gerstmann–Sträussler–Scheinker disease presenting as a subacute progressive supranuclear palsy syndrome have also been described [57].

In a retrospective study, Josephs et al. reviewed the Mayo Clinic medical records over a 93-month period and identified 22 autopsy-confirmed cases of rapidly progressive dementias with a survival period of less than 4 years [28]. Eight patients (36%) had Creutzfeldt-Jakob disease, 5 (23%) had frontotemporal lobar degeneration with motor neuron disease, 4 (18%) had a tauopathy (2 CBD and 2 progressive supranuclear palsy), 3 (14%) had dementia with Lewy bodies and 2 (9%) had Alzheimer’s disease. All patients with Creutzfeldt-Jakob disease died in less than 12 months while the patients with non-prion neurodegenerative causes of rapidly progressive dementias had a survival period of more than a year. The disease duration of both of the two CBD cases was 3.5 years and those of the two progressive supranuclear palsy cases were 1.5 years and 3.5 years [28]. Other diagnoses of rapidly progressive dementias are autoimmune and antibody-mediated encephalopathy, infectious, psychiatric, malignant, paraneoplastic, toxic-metabolic and vascular conditions [19]. Some of these conditions are potentially treatable but under-represented in autopsy-confirmed rapidly progressive dementias series [10, 19, 20, 23].

In clinical practice, many patients with neurodegenerative conditions that are typically slowly progressive are erroneously referred with a rapidly progressive dementias diagnosis. These patients are often found to have had a slow natural course over several years that has been unnoticed by family members or undiagnosed. The most common causes for rapid declines in these patients are urinary tract infection, pneumonia or an acute metabolic disturbance; and more rarely in some patients, the rapid decline is part of the natural disease course as illustrated in the present study.

The neuropathological characteristics of autopsy-confirmed CBD cases with a rapidly progressive course (RP-CBD) are not known and whether RP-CBD cases are neuropathologically distinct like other reported CBD variants warrants investigation [32–34, 41]. In this study, we have investigated these research questions by quantitatively analysing the neuropathological characteristics in six RP-CBD cases and comparing them with other CBD groups.

## Materials and methods

### Case material

As part of a large-scale pathological study on CBD, we collected 124 CBD cases from 14 UK, European, Australian and USA centres. Demographic and clinical data were obtained and reviewed by a neurologist (H.L.) while blinded to the pathological data. ‘Disease onset’ was defined as the month or year in which the patient’s family noticed the first neurological symptom that could be associated with a degenerative process and ‘disease duration’ referred to the duration from disease onset until death. Of the 124 CBD cases, 4 cases (3.2%) were identified as ‘preclinical’, who were clinically asymptomatic and had early CBD pathology. The quantitative pathological findings of three such preclinical cases have been reported [41]. The 4th preclinical CBD case was only identified in one of the collaborating centres after our study was published. Six cases from our large CBD cohort (4.8%) were identified to have a rapidly progressive clinical course (RP-CBD) with a disease duration of 3 years or less from the onset of their first clinical symptoms until reaching an advanced clinical stage. The 3-year disease duration threshold was selected as it represented the 10th percentile of a bell-shaped normal distribution. This also coincides with half of the median survival period of CBD cases in this cohort (median = 6 years). Four CBD cases (3.2%) who had developed clinical symptoms but died of another unrelated concurrent illness before reaching an advanced clinical stage were referred to as ‘intermediate’ CBD cases (Int-CBD). We hypothesise that, unlike RP-CBD, the Int-CBD cases would have had a gradual and slowly progressive illness during their entire disease course, had their clinical course not been interrupted by another illness leading to death, i.e. pulmonary embolism (Int-CBD Case 1), adenocarcinoma of the lung (Int-CBD Case 3), and myocardial infarction (Int-CBD Case 2 & 4). The remaining 110 cases (88.7%) had end-stage CBD (ES-CBD) clinically advanced disease following gradual progression of the natural disease course and died after reaching an advanced clinical stage.

To confirm the diagnosis, microscopic examination was carried out in all cases by a neuropathologist (T.R.). Selected cases were also further reviewed by two other neuropathologists (J.L.H. and E.G.). Cases with known *MAPT* mutation were excluded from the study. All cases in the RP-CBD, Int-CBD, and ES-CBD disease groups fulfilled the pathological diagnostic criteria for CBD [15]. This Queen Square Brain Bank study was approved by a London Multi-Centre Research Ethics Committee and tissue is stored for research in the Queen Square Brain Bank for Neurological Disorders under a licence from the Human Tissue Authority.

### Clinical phenotyping

Cases were assigned a clinical phenotype (corticobasal syndrome, Richardson’s syndrome, primary progressive aphasia, frontal behavioural-spatial syndrome) following the Armstrong criteria [3] whenever clinical data were sufficient, otherwise the clinical diagnosis reported by the external centres was applied as the clinical phenotype. In a large systemic literature review of 210 pathologically confirmed CBD cases, Armstrong et al. reported five main clinical phenotypes: corticobasal syndrome (37.1%), Richardson’s syndrome (23.3%), frontotemporal dementia (or frontal behavioural-spatial syndrome; 13.8%), Alzheimer’s disease-like dementia (8.1%) and primary progressive aphasia (4.8%), while others had clinical features of more than one phenotype (‘overlap’ phenotype; 5.7%) [3]. In cases with unclarified clinical diagnosis and insufficient clinical data, the phenotype is designated as ‘undetermined’. Of our 120 clinically symptomatic CBD cases, 25 cases had corticobasal syndrome [3], 16 had Richardson’s syndrome [3], 13 had primary progressive aphasia [3], 20 had frontal behavioural-spatial syndrome [3], 18 had overlap phenotypes, 2 had rapid global cognitive decline, 1 had posterior cortical atrophy [14], 4 had predominant gait disorder and/or apraxia, and 21 cases had undetermined phenotype.

### Neuropathological methods

For cases obtained from external centres, tissue slides or paraffin blocks were requested. For internal cases from the Queen Square Brain Bank (*N* = 30), the brains were divided in the mid-sagittal plane. One half, chosen randomly, was frozen, and the other half immersed and fixed in 10% buffered formalin for 3 weeks before neuropathological examination. In view of the retrospective and multi-centre nature of this study, specification of the sampling of half brain using clinical correlations such as hemisphere contralateral to the more affected hemi-body was not possible. Tissue blocks were taken using the standard Queen Square Brain Bank protocol. Eight-µm thick sections were stained with haematoxylin and eosin.

Immunohistochemistry with antibodies to the following antigens was performed using a standard avidin–biotin method: tau (AT8 clone; Thermo scientific MN1020; 1:600), 3-repeat tau and 4-repeat tau (gift from Dr Rohan de Silva; 1:750), alpha B Crystallin (Leica Biosystems NCL-ABCrys-512, clone G2JF; 1:3000), glial fibrillary acidic protein (IHC-Tek Antibody Diluent, clone GA5 mouse anti-human; 1:100), amyloid-β peptide (Biosource international, Camarillo, CA, Mouse Dako, clone 6F/3D; 1:100), transactive response DNA-binding protein 43 kDa (TDP-43; monoclonal; clone 2E2-D3; 1:2000), p62 (BD Transduction Labs, 1:200), and α-synuclein (Novocastra; 1:50).

#### Quantitative analysis of tau load

Twenty brain regions which are known to be affected in CBD and whose involvement is predicted to contribute to the clinical features were selected: the anterior frontal cortex (Brodmann area 9 or prefrontal cortex) grey matter and white matter, posterior frontal cortex (Brodmann area 4 or primary motor cortex) grey and white matter, middle temporal gyrus (Brodmann area 21) grey and white matter, superior parietal lobule (Brodmann area 7) grey and white matter, hippocampal formation (CA1-4, granular cell layer of the dentate gyrus, subiculum), amygdala, caudate, putamen, globus pallidus, subthalamic nucleus, midbrain tectum and tegmentum including substantia nigra, pontine tegmentum and base, cerebellar dentate nucleus and white matter.

Histological AT8 immunolabelled slides were digitized on a LEICA SCN400F scanner (LEICA Milton Keynes, UK) under *a* × 20 objective. Slides were viewed and managed in LEICA Slidepath (LEICA Milton Keynes, UK). Brain regions of interest were manually selected and digitally outlined (H.L. and K.D.) using Definiens Developer 2.3 (Definiens, Munich, Germany). After conversion of the red–green–blue (RGB) image to hue-saturation-density (HSD) representation [64] giving a measure of brown stain intensity at each pixel, a threshold was identified which captured the two-dimensional area of all AT8 labelled lesions (brown) in a test set of images; this same threshold setting was used for all cases. For each selected region, the ‘areal fraction’, defined by the ratio of the total area occupied by the tau-immunoreactive lesions and the entire area of interest, was computed. ‘Regional’ tau load for each brain region was expressed as a percentage (areal fraction × 100%) [24]. ‘Total’ tau load was the sum of tau load in all 20 regions. ‘Cortical’ grey matter and white matter tau load was the sum of tau load of the grey and white matter of the anterior frontal, posterior frontal, temporal and parietal regions. ‘Basal ganglia’ tau load was the sum of tau load of the caudate, putamen, globus pallidus and subthalamic nucleus.

#### Cell loss in substantia nigra

Neuronal loss in the substantia nigra pars compacta was determined in all 124 CBD cases using a four-tier semi-quantitative grading system by a neuropathologist (T.R.) from grade 0 = no neuronal loss to grade 3 = most severe neuronal loss. The substantia nigra was divided for the grading assessment into five subregions: medial, dorsomedial, dorsolateral, ventrolateral and lateral.

#### Secondary pathologies

The following additional pathologies were systematically assessed in all 124 CBD cases: p62-positive neuronal cytoplasmic inclusions seen in cases with C9orf72 expansion in the hippocampus and cerebellum, cerebral amyloid angiopathy in all cortical blocks, and AT8 and p62 for argyrophilic grains in the hippocampus and amygdala [58].

In all cases (*N* = 124), TDP-43 pathology was assessed in the hippocampus and amygdala. Additional TDP-43 staining was performed in the midbrain and pons in cases included in the subgroup analysis. Previous study by Koga et al. on TDP-43 pathology in CBD demonstrated all TDP-43 positive cases had TDP-43 pathology in either one of the four tissue sections: midbrain, subthalamic nucleus, amygdala, and pons. Since the subthalamic nucleus is not consistently sampled in some cases from external centres, tissue sections of the midbrain and pons were chosen for additional screening to ensure the presence of TDP-43 pathology especially in RP-CBD cases, was not underestimated.

The hippocampal formation was assessed for hippocampal sclerosis on haematoxylin and eosin stained histological sections [49]. To determine the level of Alzheimer’s disease neuropathological change, the ABC score was established according to the National Institute on Aging-Alzheimer’s Association Guidelines [26]. Additional sections were assessed using 3R and 4R tau immunohistochemistry, which contributed to the differentiation between Alzheimer-related (mixed 3R and 4R tau) and CBD-related neurofibrillary tangle pathology (4R tau).

### Subgroup quantitative analysis

For subgroup analysis, 12 ES-CBD cases of different clinical phenotypes (corticobasal syndrome: *N* = 4, Richardson’s syndrome: *N* = 4, frontal behavioural-spatial syndrome: *N* = 4) were selected as controls based on their age at death (mean 68.3 years ± 8.0) which matched those of the RP-CBD cases (*p* = 0.5). Only cases with well-documented clinical data were chosen. The causes of death of the 12 ES-CBD cases were septicaemia (*N* = 3), aspiration pneumonia (*N* = 3) and ‘progressive deterioration from underlying neurodegenerative condition’ (*N* = 6).

The 4 Int-CBD cases identified in our cohort were included as a comparative group because they had the same mean disease duration as the RP-CBD cases (Int-CBD: 3 years ± 1.8 vs. RP-CBD: 2.3 years ± 1.2; *p* = 0.1) but Int-CBD cases succumbed to unrelated concurrent illnesses and did not reach advanced clinical stage and were hypothesised to have an ‘interrupted’ disease course.

Comparisons of variables outlined below were performed between 6 RP-CBD, 4 Int-CBD, and 12 selected ES-CBD cases.

#### Cellular lesion types

Quantitative analysis of cellular lesion types of the RP-CBD (*N* = 6), Int-CBD (*N* = 4), and ES-CBD (*N* = 12) cases was performed using the same methods outlined in our published study on preclinical CBD cases [41].

Tau-positive cellular lesions including neuronal lesions (neurofibrillary tangles and pretangles) and astrocytic plaques were manually counted (H.L.), while neuropil threads were graded semi-quantitatively using a four-tier scale (by H.L. from grade 0 = absent to grade 3 = severe) under *a* × 20 objective in five random fields in the anterior frontal cortical (Brodmann area 9 or prefrontal cortex) grey matter and the caudate nucleus.

Coiled body pathology was not included in the quantitative analysis because we previously demonstrated that coiled bodies were much less common than other lesion types in the cortical white matter and striatum in end-stage cases [41]. Sparse coiled body pathology can be obscured in the presence of severe tau burden in the cortical white matter.

The anterior frontal cortex and caudate nucleus were chosen for this semi-quantitative analysis because these regions were identified as the earliest brain regions affected by CBD-tau pathology as observed in the preclinical CBD cases [41]. A characteristic astrocytic lesion composed of a distinct annular array of tau-positive short stubby processes with or without a visible astrocytic nucleus was included as an astrocytic plaque in manual counting.

A case lesion score (neuronal lesion score and astrocytic plaque score) for each region (anterior frontal cortex and caudate nucleus) was determined by the sum of lesion counts in all five random fields of the corresponding region. A mean lesion score was generated for each disease group (RP-CBD, Int-CBD, and ES-CBD) for each region. The ratio between mean neuronal lesion score and mean astrocytic plaque score was designated as the neuronal-astrocytic plaque ratio in the anterior frontal cortex and caudate nucleus for each disease group.

#### Neuronal loss

Neuronal loss, gliosis and microvacuolation were determined in the frontal, temporal and parietal cortical grey matter using a four-tier semi-quantitative grading system by a neuropathologist (T.R. from grade 0 = no neuronal loss/gliosis/ microvacuolation to grade 3 = most severe neuronal loss/gliosis/microvacuolation). The amount of balloon neurons identified by alpha B Crystallin immunolabelling was graded semi-quantitatively in these cortical regions (T.R. from grade 0 = no balloon neurons to grade 3 = frequent balloon neurons). Similarly, neuronal loss and gliosis were determined semi-quantitatively in the caudate, putamen, globus pallidus, subthalamic nucleus, midbrain tectum and tegmentum, pontine tegmentum and base, cerebellar dentate nucleus and gliosis in the white matter (T.R. from grade 0 = no neuronal loss and/or gliosis to grade 3 = severe neuronal loss and/or gliosis).

### Genetic analysis

DNA was extracted from frozen brain tissue (cerebellum or frontal cortex) of all 124 CBD cases. All DNA samples were sent to Gerard Schellenberg’s laboratory in University of Pennsylvania where whole exome sequencing was performed using Agilent SureSelect Capture v5 (Santa Clara, CA, USA), and was sequenced with Illumina HiSeq (San Diego, CA, USA). DNA samples of the 6 RP-CBD cases and 12 ES-CBD control cases selected for subgroup analysis also underwent genotyping using the Illumina (San Diego, CA) NeuroChip [6]. *MAPT* mutations covered by the NeuroChip were screened. Single nucleotide polymorphism imputation was carried out on the NeuroChip data using the Sanger Imputation Service to analyse for *MAPT* H1/H2 haplotype and *APOE* allele.

### Statistical analysis

The SPSS 25.0 statistical package (IBM Corporation, New York, USA) was used. Log transformation was performed to normalize data when indicated. Student’s *t* test and ANOVA (LSD post-Hoc analysis) were used to compare mean tau load (log_10_), neuronal loss (log_10_) and other continuous data. Pearson *χ*^2^ test was used to compare neuronal-to-astrocytic lesion ratios between CBD disease groups.

*P*-value of 0.05 was used as the threshold for statistical significance. To correct for multiple comparisons for regional tau load and neuronal loss analysis, a *p*-value of 0.0025 was calculated as the threshold for statistical significance (= 0.05/number of regions, i.e. 20). To correct for multiple comparisons for neuronal-to-astrocytic plaque ratio between the three disease groups (i.e. RP-CBD vs. ES-CBD, RP-CBD vs. Int-CBD and Int-CBD vs. ES-CBD), a *p*-value of 0.017 was calculated as the threshold for statistical significance (= 0.05/3). Due to the small sample sizes of the RP-CBD and Int-CBD groups, results without adjustment for multiple comparisons are also reported and discussed.

## Results

### Overview

Of the 124 cases included in our large-scale pathological study of CBD, 6 CBD cases (4.8%) had a rapidly progressive course with survival period of 3 years or less after the onset of first symptoms. The mean disease duration from onset of first symptoms until death of the RP-CBD and ES-CBD groups was 2.5 years (range 0.7–3 years; SD 0.9) and 6.7 years (range 3.5–14 years; SD 2.3), respectively (*p* < 0.001) (Table 1). There was no difference between the mean disease duration of the RP-CBD group and Int-CBD group (mean 3.0 years, range 1–5 years; SD 1.8; *p* = 0.1).

**Table 1:** Overview of demographic data of the rapidly progressive CBD (RP-CBD), intermediate-CBD (Int-CBD), and end-stage CBD (ES-CBD) groups

Disease groups	Number of cases	Gender (F:M)	Mean age at onset (years ± SD)	Mean age at death (years ± SD)	Mean disease duration (years ± SD)
Preclinical CBD	4	2:2	NA	76.5 ± 10.7	NA
RP-CBD	6	0:6	70.0 ± 12.0	72.3 ± 12.7	2.5 ± 0.9
Int-CBD	4	1:3	78.5 ± 3.3	81.5 ± 3.0	3.0 ± 1.8
ES-CBD	110	45:65	63.9 ± 7.5	70.6 ± 7.8	6.7 ± 2.3

*CBD* corticobasal degeneration, *NA* not applicable, *SD* standard deviation

The mean age at death of the Int-CBD group was 81.5 years, SD 3.0 which was significantly higher than those in the ES-CBD group (70.6 years, SD 7.8; *p* = 0.009). This finding indicates that these older Int-CBD patients had succumbed to unrelated concurrent illnesses before reaching an advanced clinical stage of CBD. Most importantly, there was no difference in the mean age at onset (*p* = 0.07) between RP-CBD and ES-CBD groups, suggesting that age was not contributory to the short survival period in the RP-CBD group. All RP-CBD cases were male whereas the female-to-male ratio of ES-CBD cases was 1:1.4 (Fisher’s exact test: *p* = 0.048).

### Rapidly progressive CBD (RP-CBD)

The demographic data of the 6 RP-CBD cases are summarised in Table 2. Their disease duration ranged from 8 months to 3 years. Two presented with rapid global cognitive decline, and others presented with either rapidly progressive corticobasal syndrome (*N* = 2) or Richardson’s syndrome (*N* = 2). Prion disease was considered as a differential diagnosis in 3 cases; one underwent ante-mortem brain biopsy (RP-Case 4) and the autopsies of the other 2 cases (RP-Case 2 & 6) were performed in a mortuary accredited for carrying out post-mortem examinations on high-risk cases. Histological examination including negative immunohistochemistry staining of pathological prion protein had ruled out prion disease as the underlying diagnosis in all 6 cases.

**Table 2:** Demographic data of six rapidly progressive CBD (RP-CBD) cases

Case no	Clinical phenotype	Gender	Age at onset (years)	Age at death (years)	Duration (years)	Antemortem clinical diagnosis of prion disease	Causes of death
RP-Case 1	CBS	Male	80	83	3	No	Bronchopneumonia
RP-case 2	RS	Male	56	59	2.6	Yes	Cardiorespiratory failure
RP-case 3	GCD	Male	71	73	2	No	Bronchopneumonia
RP-case 4	GCD	Male	57	57	0.7	Yes	Respiratory insufficiency
RP-case 5	CBS	Male	70	73	3	No	Cardiorespiratory failure
RP-case 6	RS	Male	86	89	3	Yes	Cardiorespiratory failure

*CBD* corticobasal degeneration, *CBS* corticobasal syndrome, *GCD* global cognitive decline, *RP-CBD* rapidly progressive CBD, *RS* Richardson’s syndrome, *SD* standard deviation

We outlined below the clinical summary of RP-Case 2 as a case illustration. This patient was diagnosed and seen by our group (H.L.) in the specialist movement disorders clinic at the National Hospital for Neurology and Neurosurgery, Queen Square in London and was reviewed frequently throughout the disease course until death due to his rapid deterioration.

### Case illustration (RP-case 2)

This patient presented to a specialist movement disorders clinic with an 18-month history of progressive dysarthria, generalised bradykinesia, impaired manual dexterity, hypersalivation, freezing of gait and frequent backward falls. His children remarked on his impulsivity and poor insights of his motor impairment and personality changes. The mild improvements in his motor symptoms with Levodopa therapy were only transient. Examination revealed a staring gaze with decreased blink rate, vertical supranuclear gaze palsy, upright posture, nuchal and limb rigidity, and postural instability. Magnetic resonance imaging performed 19 months after symptom onset showed moderate midbrain atrophy and generalised atrophy. Dopamine transporter scan showed markedly reduced tracer uptake. Neuropsychometric testing revealed dysfunction in the anterior, subcortical and nondominant posterior regions. An initial diagnosis of progressive supranuclear palsy was made.

Over the next 12 months, his motor and cognitive functions deteriorated rapidly, and he required assistance for most of his daily activities. He was disinhibited verbally and physically; he became hypersexual and developed stereotypy behaviours such as fiddling with a key ring and counting purposelessly. He had daily backward falls resulting in multiple head injuries and fractures. He was incontinent and had significant weight loss due to worsening dysphagia and frequent aspiration. He died 31 months after the onset of his first symptoms.

## Quantitative analysis of tau load

### Total, cortical and basal ganglia tau load

**Fig. 1:**
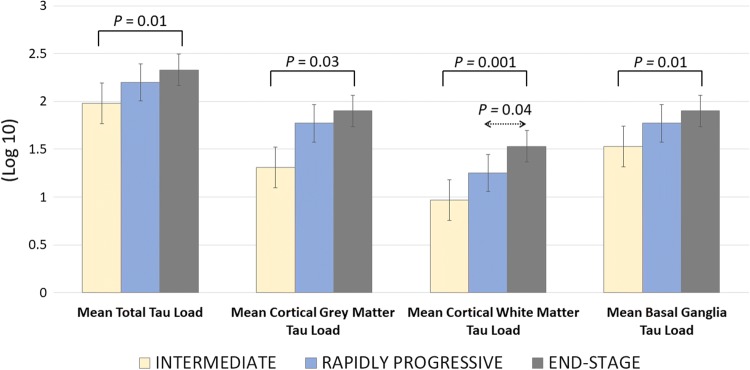
Mean Total, Cortical Grey Matter, Cortical White Matter and Basal Ganglia Tau Load of the Intermediate (Int-CBD), Rapidly Progressive (RP-CBD) and End-Stage (ES-CBD) Groups. All the illustrated tau load measurements of the intermediate group were significantly less than the end-stage group (ANOVA, LSD post-Hoc analysis; *p* < 0.05). All tau load measurements were the same statistically between the rapidly progressive and end-stage CBD groups, except there was less cortical white matter tau load in the rapidly progressive group than in the end-stage group (ANOVA, LSD post-Hoc analysis; *p* = 0.04). Error bars represent one standard error of the mean (SEM)

The mean total tau load, cortical grey matter tau load and basal ganglia tau load did not differ between the RP-CBD and the ES-CBD groups (*p* > 0.05, Fig. 1). The cortical white matter tau load of the RP-CBD group was less than that of the ES-CBD group (*p* = 0.04). On the other hand, the mean total tau load, cortical grey and white matter tau load and basal ganglia tau load of the Int-CBD group were all less than that of the ES-CBD group (*p* < 0.03).

### Regional tau load

**Fig. 2:**
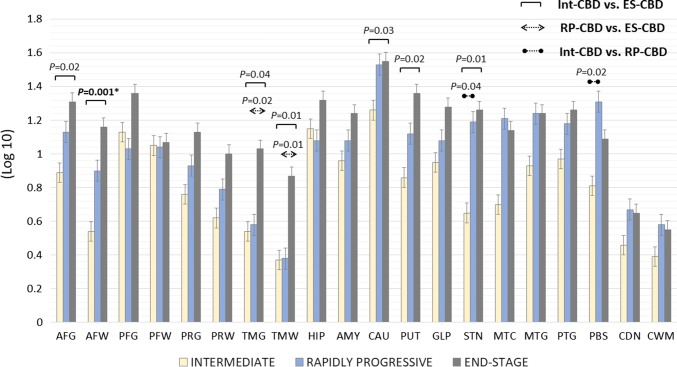
Mean Regional Tau Load of the Intermediate (Int-CBD), Rapidly Progressive (RP-CBD) and End-Stage (ES-CBD) Groups. Mean regional tau load in the anterior frontal grey and white matter, temporal grey and white matter, caudate, putamen, and subthalamic nucleus were significantly less in the Int-CBD group than in the ES-CBD group (ANOVA, LSD post-Hoc analysis; *p* < 0.05). Mean regional tau load in the temporal grey and white matter was significantly less in the RP-CBD group when compared with the ES-CBD group (ANOVA, LSD post-Hoc analysis; *p* < 0.05). *Mean regional tau load in the anterior frontal white matter was the only region showing significant difference between the Int-CBD and ES-CBD group after adjustment for multiple comparisons (*p* = 0.001). Error bars represent one standard error of the mean (SEM). *AFG *anterior frontal grey matter, *AFW* anterior frontal white matter, *AMG* amygdala, *CAU* caudate, *CDN* cerebellar dentate nucleus, *CWM* cerebellar white matter, *GLP* globus pallidus, *HIP* hippocampus, *MTC* midbrain tectum, *MTG* midbrain tegmentum, *PBS* pontine base, *PFG* posterior frontal grey matter, *PFW* posterior frontal white matter, *PRG* parietal grey matter, *PRW* parietal white matter, *PTG* pontine tegmentum, *PUT* putamen, *STN* subthalamic nucleus, *TMG* temporal grey matter, *TMW* temporal white matter

Regional tau load of the 20 selected brain regions was determined (Fig. 2). The tau load was the same between the RP-CBD and ES-CBD groups in the majority of the regions analysed, except in the temporal grey (*p* = 0.02) and white matter (*p* = 0.01) where the regional tau load was less in the RP-CBD group. When comparing regional tau load between the Int-CBD and ES-CBD groups, the tau load in all regions was numerically less in the Int-CBD group. Tau load in the following regions was less in the Int-CBD group than in the ES-CBD group with statistical significance (*p* < 0.05): anterior frontal grey and white matter, temporal grey and white matter, caudate, putamen, and subthalamic nucleus. After adjustment for multiple comparisons, the anterior frontal white matter was the only region that showed statistical significance when comparing regional tau load between Int-CBD and ES-CBD groups (*p* = 0.02).

## Cell loss in substantia nigra

**Fig. 3:**
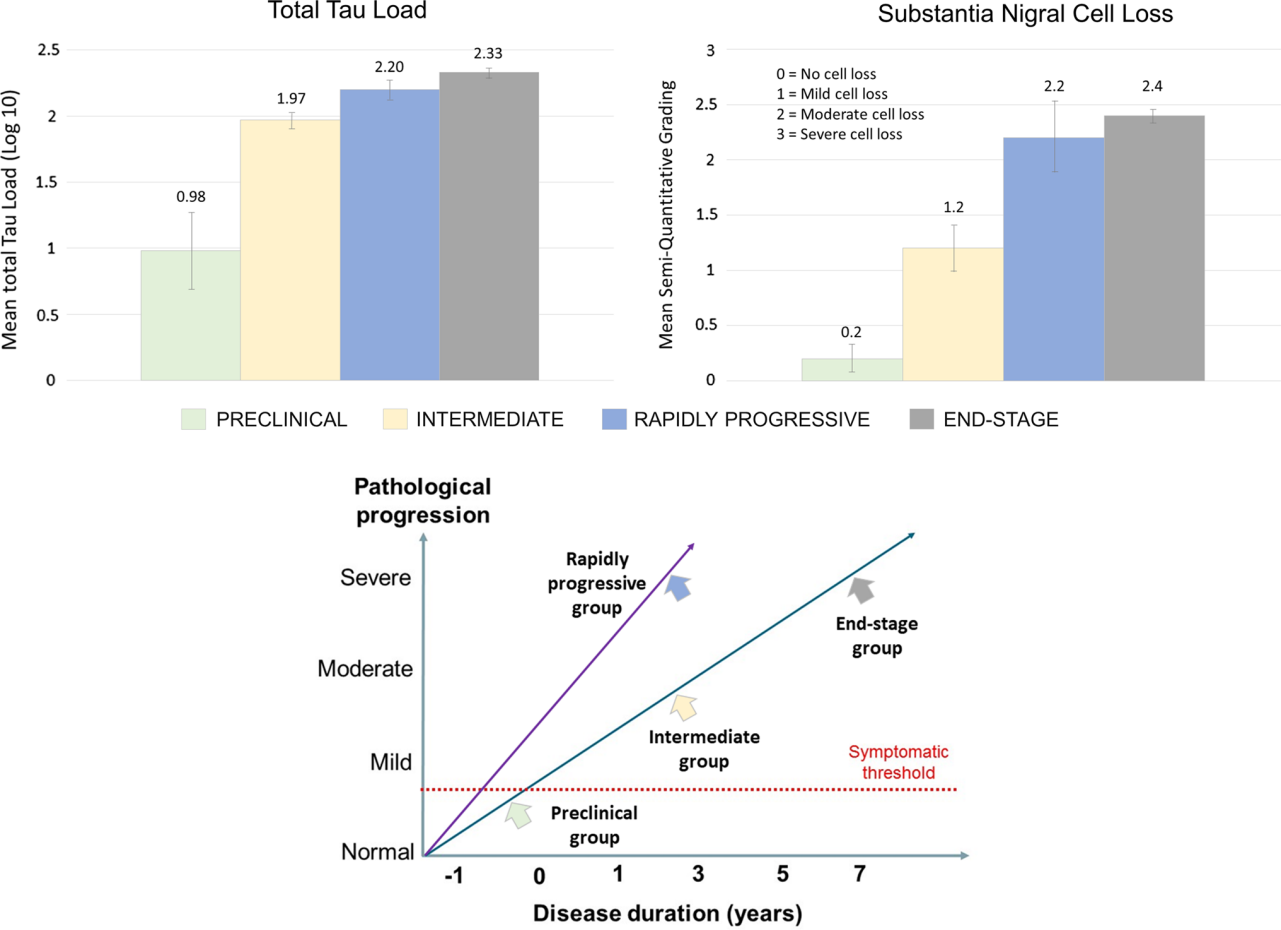
Proposed Pathological Progression of Different CBD Disease Groups. Top panels illustrate increasing total tau load and severity of nigral cell loss as the disease progresses from preclinical to intermediate, and to end-stage disease. No significant differences in total tau load and severity of nigral cell loss were identified between the rapidly progressive and end-stage groups. The bottom panel illustrates that the preclinical, intermediate and end-stage groups probably follow the same trajectory of disease progression based on our quantitative data above, whereas the rapidly progressive group demonstrates a more aggressive disease course with their pathological changes reaching the same level of severity as the end-stage group within 3 years or less after symptom onset. Notably, the mean disease duration of the Int-CBD and RP-CBD groups did not differ statistically but the severity of pathological changes in the Int-CBD group were significantly milder

Semi-quantitative assessment of cell loss using a four-tier grading system showed that the mean nigral cell loss in the RP-CBD (*N* = 6) and ES-CBD (*N* = 110) groups was moderate (grade 2) to severe (grade 3) in all five substantia nigra subregions and there was no significant difference between the two groups (*p* > 0.3; Fig. 3). In contrast, the mean cell loss in the Int-CBD group was only mild (grade 1) in all nigral subregions and was significantly less severe in all subregions when compared with the ES-CBD group (*p* < 0.03). After adjusting for multiple comparisons, the mean cell loss in the medial and dorsomedial subregions remained significantly milder in the Int-CBD group than the ES-CBD group (*p* < 0.001). Similarly, after adjusting for multiple comparisons, cell loss in the medial nigral subregion was significantly less severe in the Int-CBD group than in the RP-CBD group (*p* < 0.01).

## Secondary pathologies

No difference was found in the presence of hippocampal sclerosis (RP-CBD: 1 in 6 (17%), ES-CBD: 10 in 110 (9%); *χ*^2^ test: *p* = 0.46), argyrophilic grain disease (RP-CBD: 3 in 6 (50%), ES-CBD: 70 in 110 (64%); *χ*^2^ test: *p* = 0.67) between RP-CBD and ES-CBD cases. Mild hippocampal TDP-43 pathology was observed in only two RP-CBD cases (Case 5: corticobasal syndrome phenotype and Case 6: Richardson’s syndrome). Of the 11 cases with hippocampal sclerosis, nine had TDP-43 pathology inclusive of a RP-CBD case (RP-Case 5). No difference in the severity of Aβ Thal phase, Braak and Braak neurofibrillary tau stage, CERAD neuritic plaque score and cerebral amyloid angiopathy was identified between RP-CBD and ES-CBD cases (Mann Whitney test; *p* > 0.4). *C9orf72* expansion mutation-related inclusions were not identified in any of the 124 cases in the present cohort.

Secondary pathologies may influence the phenotypic presentation and disease course of neurodegenerative tauopathies [29, 32, 34, 43, 60, 67]. Therefore, it is noteworthy that concomitant pathologies were not contributary to the rapid progression in RP-CBD (Table 3).

**Table 3:** Secondary pathologies and genetic data in the six rapidly progressive CBD (RP-CBD) cases

Case no	Hippo-campal sclerosis	Grains*	TDP-43**	CAA***	Thal phase****	Braak and braak NFT Stage	ABC score (level of AD change)#	*MAPT* H1/H2 haplotype	Known pathogenic *MAPT* mutations	*APOE* allele
RP-case 1	No	2	0	1	2	II	A1, B1, C1 (low)	H1/H2	Nil	E3/E3
RP-case 2	No	0	0	0	0	I	A0, B1, C0 (not)	H1/H1	Nil	E3/E3
RP-case 3	No	1	1 (P)	0	2	0	A1, B0, C2 (low)	H1/H1	Nil	E3/E3
RP-case 4	No	1	0	0	0	II	A0, B1, C0 (not)	H1/H1	Nil	E3/E3
RP-case 5	Yes	0	1 (H, A)	0	0	III	A0, B2, C0 (not)	H1/H1	Nil	E3/E4
RP-case 6	No	0	1 (H, A)	3	5	IV	A3, B2, C3 (intermediate)	H1/H1	Nil	E3/E3

*A* amygdala, *H* hippocampus, *P* pons

*Argyrophilic grain stages applied [58]

**CAA: cerebral amyloid angiopathy (Grading applied: none = 0, mild = 1, moderate = 2, frequent = 3)

***TDP-43: TAR DNA-binding protein of 43 kDa (Grading applied: none = 0, mild = 1, moderate = 2, frequent = 3)

****Thal Aβ phase

#National Institute on aging – Alzheimer Association (NIA-AA) ABC score (level of Alzheimer’s disease related neuropathological change) [26]

### TDP-43 pathology

No difference was identified in the presence of TDP-43 pathology (RP-CBD: 3 in 6 (50%), ES-CBD: 29 in 110 (26%); Fisher’s exact test: *p* = 0.35) between RP-CBD and ES-CBD cases.

Among the three TDP-43 positive RP-CBD cases, two cases had mild TDP-43 pathology in the hippocampus and amygdala (RP-5, RP-6) and one case had mild TDP-43 pathology in the pontine tegmentum (RP-3), who presented with rapid global cognitive decline without any record of having a downgaze palsy. CBD cases with a Richardson’s syndrome phenotype was not more common in the positive TDP-43 or severe TDP-43 groups (χ^2^/Fisher’s exact test: *p* > 0.5).

Interestingly, tau burden in the olivopontocerebellar system was more severe in the TDP-43 positive group (*p* = 0.02), consistent with the study findings by Koga et al. [32].

## Subgroup quantitative analysis

For subgroup analysis, 12 ES-CBD cases were selected as age-matched controls. Quantitative analysis on cellular lesion type and neuronal loss were compared between the RP-CBD (*N* = 6), Int-CBD (*N* = 4) and ES-CBD (*N* = 12) groups. No difference was found in the presence/severity of TDP-43 pathology between the RP-CBD, Int-CBD, and ES-CBD groups in the subgroup analysis (*p* = 0.28).

### Cellular lesion types

Quantitative analysis was performed to study the lesion types affected by tau pathology in the anterior frontal cortex and caudate. RP-CBD cases had more neuropil thread pathology in the caudate than that of ES-CBD cases with borderline significance (*p* = 0.05), while no difference in neuropil thread pathology was found in the anterior frontal cortex between the two groups (*p* = 0.47). Numerically, the RP-CBD group had the highest mean neuronal lesion score (anterior frontal cortex: 73, caudate: 39) followed by the ES-CBD (anterior frontal cortex: 70, caudate: 26) and Int-CBD groups (anterior frontal cortex: 47, caudate: 33). As for astrocytic plaques, the Int-CBD group had the highest mean score (anterior frontal cortex: 33, caudate: 29), followed by the ES-CBD group (anterior frontal cortex: 23, caudate: 17); RP-CBD group had the least number of astrocytic plaques (anterior frontal cortex: 10, caudate: 7).

**Fig. 4:**
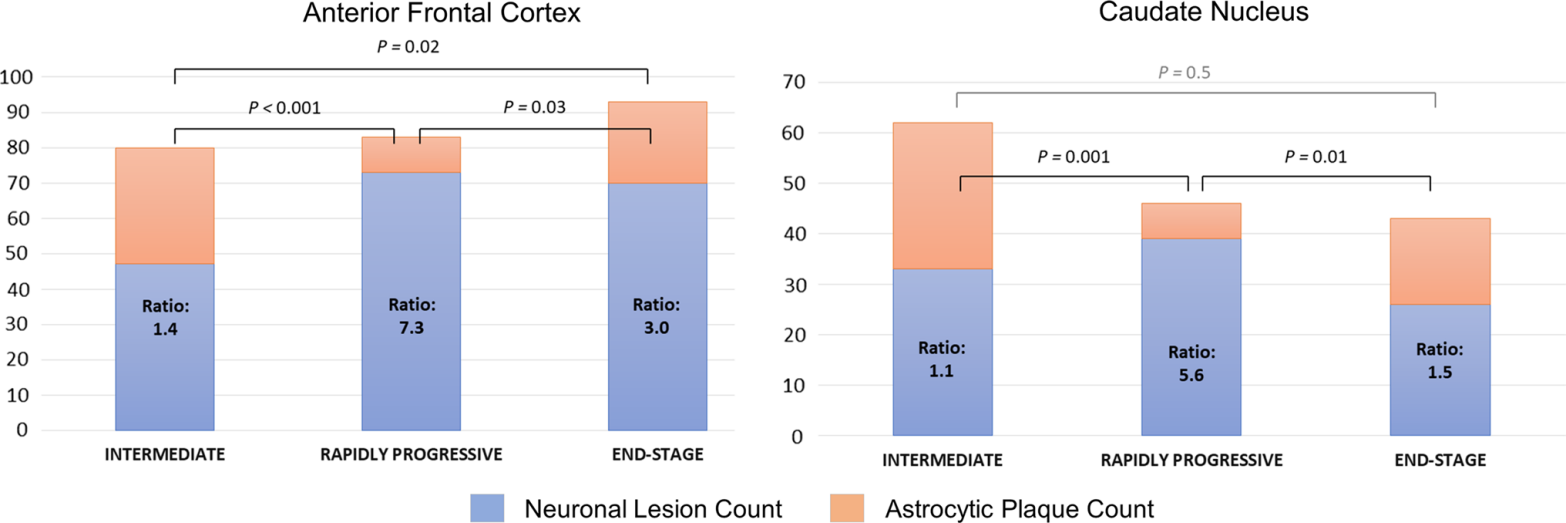
Neuronal-To-Astrocytic Plaque Ratios of the Intermediate (Int-CBD), Rapidly Progressive (RP-CBD) and End-Stage (ES-CBD) Groups of the Anterior Frontal Cortex and Caudate Nucleus. The ratios for both regions in the RP-CBD group were significantly greater than the Int-CBD and ES-CBD groups, indicating proportionally higher number of neuronal lesions (neurofibrillary tangles and pretangles) than astrocytic plaques in RP-CBD cases

The neuronal-to-astrocytic plaque ratios of the RP-CBD group in the anterior frontal cortex (7.5) and caudate (5.6) were significantly greater than those of the ES-CBD (anterior frontal cortex: 3.0, caudate: 1.5) and Int-CBD groups (anterior frontal cortex: 1.4, caudate: 1.1) (*p* < 0.05; Fig. 4). These findings indicate that the RP-CBD group had a proportionally higher number of neuronal lesions than astrocytic plaques when compared with the ES-CBD (*χ*^2^ test; anterior frontal cortex: *p* = 0.03, caudate; *p* = 0.01) and Int-CBD groups (*χ*^2^ test; anterior frontal cortex: *p* < 0.001, caudate; *p* = 0.001). When compared between the ES-CBD and Int-CBD groups, the neuronal-to-astrocytic plaque ratio in the anterior frontal cortex was greater in ES-CBD than in Int-CBD in the anterior frontal cortex (*χ*^2^ test; *p* = 0.02), but no difference was found in the caudate (*χ*^2^ test; *p* = 0.46). Following correction for multiple comparisons, statistical significance was achieved for RP-CBD vs. ES-CBD in the caudate and RP-CBD vs. Int-CBD in both regions. Among the ES-CBD and Int-CBD cases, there was a significant positive correlation between neuronal-to-astrocytic plaque ratio and disease duration in the anterior frontal cortex and caudate (both regions: Pearson’s coefficient = 0.6; *p* = 0.02). No correlation was identified in the RP-CBD cases. These findings suggest a progressive increase in neuronal-to-astrocytic plaque ratio in both examined regions as the disease advances.

### Neuronal loss

Semi-quantitative assessment of neuronal loss, gliosis, microvacuolation and the amount of balloon neurons was performed in anterior frontal, temporal and parietal cortical regions in the RP-CBD, Int-CBD, and ES-CBD groups. No differences in these parameters were identified in these cortical regions between the three groups, except that neuronal loss in the parietal cortex was more severe in the ES-CBD group than in the RP-CBD group (*p* = 0.049).

Similarly, neuronal loss and gliosis in other selected brain regions were analysed semi-quantitatively. These parameters did not differ significantly between the three groups in the following regions: caudate, putamen, globus pallidus, subthalamic nucleus, midbrain tectum and tegmentum, pontine tegmentum and base, cerebellar dentate nucleus and white matter. The only significant difference identified was that gliosis in the caudate nucleus was more severe in the ES-CBD group than in the Int-CBD group (*p* = 0.02). After adjusting for multiple comparisons, no significant difference in these variables was found when compared between the three groups.

## Genetic analysis

No known pathogenic mutations of the *MAPT* gene were identified in the present cohort of 124 pathologically confirmed CBD cases including the six RP-CBD cases. A significantly greater proportion of ES-CBD cases (H1H1: *N* = 4, H1H2: *N* = 8) carried the H2 haplotype, known to be a protective factor, when compared with the RP-CBD group (H1H1: *N* = 5, H1H2: *N* = 1; Fisher’s exact: *p* < 0.001) [9, 25]. There was no difference in the distribution of *MAPT* H1/H1 haplotype between TDP-43 positive or negative group (χ^2^: *p* = 0.26). *APOEɛ4* allele was identified in one case in each of the RP-CBD (Case 5) and ES-CBD groups (Fisher’s exact: *p* = 0.55).

## Discussion

Of the 124 CBD cases included in our CBD neuropathological study, 6 (4.8%) had well-documented evidence of a rapidly progressive clinical course and reached an advanced clinical stage within 3 years or less after the first symptom onset. These RP-CBD cases had advanced pathological changes as demonstrated by the severity of their overall tau burden and nigral cell loss. Neuronal tau lesions rather than astrocytic plaques were predominant in the RP-CBD variant indicating a distinct cellular vulnerability that differs from other CBD disease subgroups. No other factors such as age at onset, concomitant pathologies or pathogenic tau mutations were found to be contributory to the rapid progressive course of these RP-CBD cases. Our findings suggest that RP-CBD is a distinct fulminant CBD variant that has an aggressive disease process resulting in rapid progression both clinically and pathologically.

### Tau burden

The level of pathological tau burden is a good measure of the neurodegenerative process in both Alzheimer’s disease and primary tauopathies [8, 22]. Despite the significantly shorter disease duration, RP-CBD cases had the same level of tau burden as those in the ES-CBD group. While tau burden in the cortical grey matter and basal ganglia was similar in the two groups, cortical white matter tau load was less in the RP-CBD group, which suggests that CBD-tau accumulation in the cortical white matter may lag behind tau accumulation in other brain regions in RP-CBD cases. Region-to-region comparison showed that tau burden in the 20 selected brain regions was the same in the RP-CBD and ES-CBD groups, except in the temporal cortical grey and white matter where the amount of tau was significantly less in the RP-CBD group. This suggests that the temporal cortex is either relatively preserved in RP-CBD or that it is one of the latest regions to be severely affected by CBD-tau pathology in RP-CBD. Within the basal ganglia circuitry, the highest regional tau load was identified in the caudate, putamen, globus pallidus and subthalamic nucleus in the ES-CBD group, and RP-CBD cases also had the same level of tau burden in these severely affected regions. Brain regions identified to have relatively less tau burden when compared with ES-CBD (i.e. cortical white matter and temporal cortical grey and white matter) most likely did not play a primary role in contributing to the rapid clinical deterioration in RP-CBD. In other words, these regions may be considered as ‘non-strategic’ regional correlates, contrasting with the frontal and parietal cortices and basal ganglia circuitry which are ‘strategic’ brain regions where a comparable extent of severe tau burden was identified in both RP-CBD and ES-CBD groups. The hypothesis of the temporal lobe being a non-strategic region may be further corroborated in other neurodegenerative conditions. For instance, semantic variant of primary progressive aphasia, characterised by predominant temporal lobe involvement and TDP-43 Type C pathology, tends to have longer disease duration and is considered a more benign form of frontotemporal lobar degeneration [37, 46].

### Cell loss in the substantia nigra

Studies by our group and others have shown that the substantia nigra is typically preserved in early CBD cases and nigral cell loss becomes more severe as the disease progresses [41, 50, 51, 53]. Again, despite the shorter mean disease duration, RP-CBD cases had the same level of nigral cell loss as ES-CBD, while Int-CBD had milder nigral cell loss than both RP-CBD and ES-CBD.

### Preclinical CBD

**Fig. 5:**
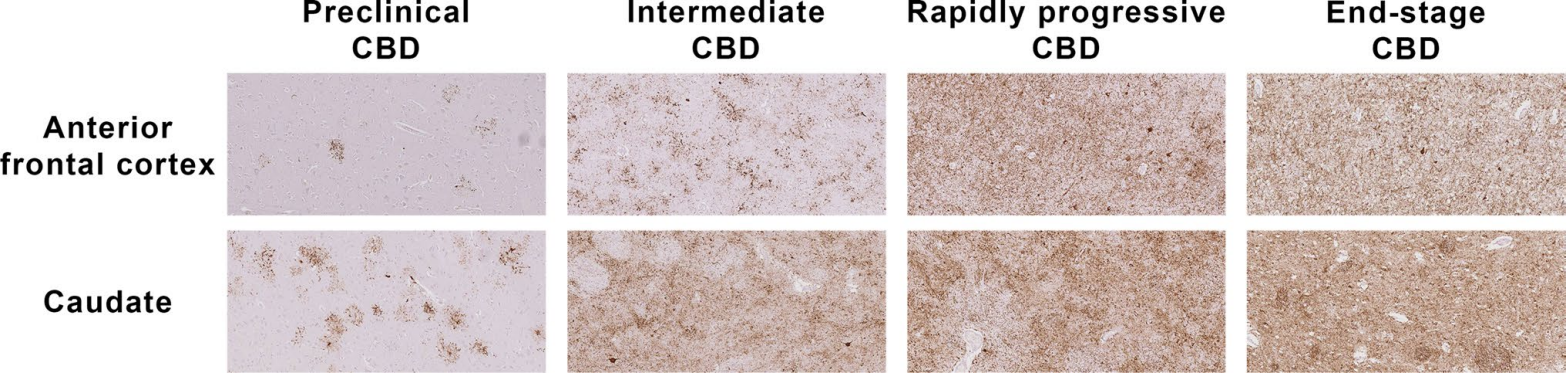
Tau Immunohistochemistry (AT8) Sections of Preclinical (Case 1; Ling et al. Brain 2016), Intermediate and Rapidly Progressive (RP-Case 4) and End-Stage CBD (ES-Case 11) Cases (× 10 objective)

Previously, we reported the neuropathological characteristics of three clinically asymptomatic cases who were found to have early CBD pathology at post-mortem [41]. These preclinical CBD cases provided valuable insights into the regional, neural network and cellular vulnerability at the earliest disease process of CBD. Although their overall tau burden was nine times less than that of the ES-CBD cases (Fig. 5), tau aggregates were already widespread in brain regions typically affected in ES-CBD [41]. The substantia nigra in preclinical cases was preserved [41] contrasting with the moderate to severe nigral cell loss in ES-CBD (Fig. 3). Additional analysis also showed that Int-CBD had more severe regional nigral cell loss than preclinical CBD (*N* = 4) with borderline significance in 4 of 5 subregions studied (medial: *p* = 0.08, dorsomedial: *p* = 0.08, dorsolateral: *p* = 0.07 and ventrolateral: *p* = 0.07). Astrocytic plaques were the predominant tau lesion in preclinical CBD suggesting that CBD may begin as a primary astrogliopathy although neuronal lesions eventually predominate as the disease process advances [41]. This proposed shift of cellular vulnerability to tau accumulation with disease progression is also supported by our findings of the higher neuronal-to-astrocytic plaque ratio in ES-CBD (3.1) than in Int-CBD (1.4) in anterior frontal cortex (Fig. 4).

Increasing evidence suggests that glial activation and chronic neuroinflammation play a causal role in ageing and neurodegeneration [39]. Activated astrocytes and microglia and elevated proinflammatory markers can be demonstrated in tauopathies [54]. Thorn-shaped astrocytes commonly observed in the brains of cognitively normal elderly and primary tauopathies are collectively referred to as age-related tau astrogliopathy (ARTAG) [36]. Whether the primary role of astrocytic tau in ARTAG and preclinical CBD is pathogenic or protective remains elusive. Nevertheless, these observations indicate that glial cells may be a useful target for early diagnosis using neuroimaging and molecular-based therapy [54].

### Intermediate-CBD

In the present series, we included an ‘intermediate’ CBD group (Int-CBD). These Int-CBD patients had developed CBD and, because of their older age, were at higher risk of having other medical conditions such as malignancy or ischaemic heart disease and had succumbed to these illnesses before reaching an advanced clinical stage of CBD. We hypothesised that their pathological progression was ‘interrupted’ and that their pathological features most likely represent an ‘intermediate’ disease stage. These four Int-CBD cases were included in the present study as a ‘duration-control’ group as they had the same mean disease duration as RP-CBD. Comparing the findings of the Int-CBD, RP-CBD, and ES-CBD groups allowed us to conclude that the features of RP-CBD were truly their distinct pathological substrates rather than shorter disease duration. We demonstrated the tau burden in all brain regions was less severe in Int-CBD than in ES-CBD group (Fig. 2). The mean nigral cell loss in the Int-CBD group was milder (grade 1) in all nigral subregions than RP-CBD and ES-CBD, both of which demonstrated moderate (grade 2) to severe (grade 3) nigral cell loss (Fig. 3). Overall, these findings indicated that the pathological changes of the preclinical, Int-CBD, ES-CBD cases followed the same trajectory of disease progression and that this differed from the distinctly aggressive disease course of RP-CBD (Fig. 3).

### Cortical cell loss, gliosis, and microvacuolation in CBD are time-dependent changes

Neuropathological examination of neuronal loss, gliosis, and microvacuolation demonstrated a range of severity in the Int-CBD, RP-CBD, and ES-CBD groups. In some ES-CBD cases, the frontal and parietal cortices showed advanced changes exceeding the severity of those observed in Int-CBD. In preclinical CBD cases, no cell loss or gliosis was seen in the frontal, parietal and temporal cortices, striatum and subthalamic nucleus [41].

Based on our observations, we suggest that the neuronal loss, gliosis and microvacuolation are time-dependent changes. Their regional distribution and severity are also phenotype-dependent, as evidenced by the findings of previous clinicopathological and neuroimaging studies [35]. For instance, corticobasal syndrome phenotype demonstrates more extensive frontal and parietal cortical neuronal loss when compared with RS phenotype [33].

In our subgroup analysis, 12 ES-CBD cases of different clinical phenotypes (corticobasal syndrome: *N* = 4, Richardson’s syndrome: *N* = 4, frontal behavioural-spatial syndrome: *N* = 4) were selected as controls for the subgroup analysis but the number of cases representing each phenotype remained small. The lack of significant statistical difference in these parameters between the Int-CBD, RP-CBD and ES-CBD groups may be due to their small sample sizes.

### Distinct cellular vulnerability of RP-CBD

Since the tau load varies between regions, cases and disease groups, we devised the neuronal-to-astrocytic plaque ratio as a measure of the relative frequency and distribution of the cellular lesion types. The anterior frontal cortex and caudate nucleus, the two regions proposed to be the earliest regions affected by CBD-tau pathology [41], were selected for quantitative subgroup analysis. We previously demonstrated that astrocytic plaques predominated the preclinical cases in these two regions in contrast to ES-CBD cases, in which neuronal tau lesions were predominant [41]. In the present study, the positive correlation between neuronal-to-astrocytic plaque ratios and disease duration demonstrated among the ES-CBD and Int-CBD cases in both the anterior frontal cortex and caudate further corroborates the notion that neuronal tau lesions become more predominant in relation to astrocytic plaques as the disease progresses.

Intriguingly, cellular vulnerability to abnormal tau accumulation in RP-CBD is distinctly different from other disease groups and in a manner opposite to preclinical cases. The neuronal-to-astrocytic plaque ratio in both the anterior frontal cortex and caudate nucleus was significantly higher in RP-CBD (anterior frontal: 7.5, caudate: 5.6) than in Int-CBD (anterior frontal: 1.4; *p* < 0.001) (caudate: 1.1; *p* = 0.001) and ES-CBD (anterior frontal: 3.0; *p* = 0.03) (caudate: 1.5; *p* = 0.01). This finding indicates that neuronal lesions were the predominant tau lesion type in the RP-CBD group with 5–7 times more neuronal lesions than astrocytic plaques present in the examined regions. While the total tau burden and neuronal loss were similar between the RP-CBD and ES-CBD groups, RP-CBD had the highest mean neuronal lesion score. Accumulation of hyperphosphorylated tau in neurons leads to neuronal dysfunction and eventually neuronal death [5]. Previous studies in Alzheimer’s disease and primary tauopathies demonstrated a dose-dependent clinico-pathological correlation between neuronal tau accumulation and clinical dysfunction [5, 7, 40, 66]. Abnormal tau accumulation in selectively vulnerable neurons in RP-CBD probably cause accelerated neuronal dysfunction. We hypothesise that the predominant neuronal tau lesions observed in RP-CBD most likely contributed as the pathological substrate of their aggressive clinical disease course.

### Is RP-CBD a distinct aggressive variant of CBD?

The concept of different ‘prion-like strains’ of tau aggregates has been consolidated by the demonstration that the inoculation of transgenic tau mice with various tau strains causes strain-specific intracellular pathology in distinct cell types and brain regions following specific neural network connections as well as inducing different rates of network propagation [2, 12, 21, 22, 59]. This concept is further underpinned by recent cryo-electron microscopy studies demonstrating distinct conformers of assembled tau are responsible for different tauopathies [16-18]. Furthermore, Kaufman et al. suggested that strains alone were accountable for the diverse neuropathological presentation of human tauopathies [30]. The potential association between strains and clinical progression of neurodegenerative diseases is also described in other misfolded proteins such as amyloid-β. A study of rapidly progressive Alzheimer’s disease cases reported a correlation with the levels of amyloid-β_42_ with distinct structural characteristics, suggesting a possible mechanistic link between conformers and their aggressive clinical disease course [13].

Our systemic analysis suggests that the preclinical, Int-CBD and ES-CBD groups most likely follow the same trajectory of disease progression, while RP-CBD is a distinct variant characterised by rapid clinical and pathological progression (Fig. 3). It seems reasonable to hypothesise that RP-CBD cases are associated with a specific tau ‘strain’, which governs their rapid disease process resulting in the development of advanced neuropathological changes within a very short timeframe. Further investigations are warranted to confirm this hypothesis.

CBD with severe TDP-43 pathologies was also proposed as a distinct CBD variant characterized by a Richardson’s syndrome clinical phenotype and severe tau pathology in the olivopontocerebellar system [32, 34, 62, 67]. Likewise, the findings of the present series also showed that the presence of TDP-43 pathology was associated with greater tau burden in the olivopontocerebellar system. Importantly, mild TDP-43 pathology was observed in 3 of the 6 RP-CBD cases and the proportion of TDP-43 positive cases did not differ from those in ES-CBD (29 out of 110 cases). We can conclude that the aggressive disease course of the RP-CBD variant could not have been attributed to additional TDP-43 pathology. Moreover, we did not identify any significant difference in other secondary pathologies between the RP-CBD and ES-CBD disease groups.

The clinical phenotypes of the six RP-CBD cases were not limited to one specific phenotype: two cases had rapid global cognitive decline, two had corticobasal syndrome and the other two had Richardson’s syndrome. The spectrum of clinical phenotypes in RP-CBD indicates that selective vulnerability in this distinct CBD variant involves a range of neural networks. Increasing evidence indicates that the molecular basis for selective vulnerability underlying clinical phenotypes may be determined by differences in tau strains and, in part, genetic variants [27, 59]. It is noteworthy that all 6 RP-CBD cases were male. *MAPT* H2 haplotype is known to be protective and was found in only one RP-CBD case [9, 25], which is likely to be one of the contributing factors to the aggressive disease process in RP-CBD. An intronic variant of the tripartite motif-containing protein 11 (*TRIM11*) has been linked to the classic and more aggressive phenotype of progressive supranuclear palsy, Richardson’s syndrome [27]. *TRIM11* is predominantly expressed in the neurons in the basal ganglia and cerebellum [27]. Whether *TRIM11* is also a genetic modifier of CBD phenotypes and variants such as RP-CBD should be the subject of future research.

## Conclusions

This is the first study to report the pathological substrates of RP-CBD. We propose that fulminant CBD is a distinct clinicopathological CBD variant, likely to be associated with a specific tau ‘strain’. RP-CBD comprised 5% of our CBD cohort. The identification of rapid progressors is particularly important for therapeutic trials as their skewed representation in the treatment arms may potentially confound the interpretation of treatment outcome [61]. Two distinct patterns of decline, fast and slow, were reported in Alzheimer’s disease [61], which has been posed as a challenge in the quest for an effective disease-modifying treatment [11, 44]. The recent finding of a positive correlation between neurofilament light chain level in cerebrospinal fluid and disease progression rate in progressive supranuclear palsy, Alzheimer’s disease, dementia with Lewy bodies and frontotemporal lobar degeneration [1, 56] is a promising start to understand the underlying pathophysiology, and to identify accurate biomarkers and genetic modifiers of rapid progressors in neurodegenerative diseases such as fulminant CBD.

## Abbreviations

CBD: Corticobasal degeneration
ES-CBD: End-stage corticobasal degeneration
Int-CBD: Intermediate corticobasal degeneration
SD: Standard deviation
TDP-43: Transactive response DNA-binding protein 43 kDa
